# Anisotropic magnetocaloric effect in Fe_3−*x*_GeTe_2_

**DOI:** 10.1038/s41598-019-49654-4

**Published:** 2019-09-13

**Authors:** Yu Liu, Jun Li, Jing Tao, Yimei Zhu, Cedomir Petrovic

**Affiliations:** Condensed Matter Physics and Materials Science Department, Brookhaven National Laboratory, Upton, New York 11973 USA

**Keywords:** Ferromagnetism, Magnetic properties and materials

## Abstract

We present a comprehensive study on anisotropic magnetocaloric porperties of the van der Waals weak-itinerant ferromagnet Fe_3−*x*_GeTe_2_ that features gate-tunable room-temperature ferromagnetism in few-layer device. Intrinsic magnetocrystalline anisotropy is observed to be temperature-dependent and most likely favors the long-range magnetic order in thin Fe_3−*x*_GeTe_2_ crsytal. The magnetic entropy change Δ*S*_*M*_ also reveals an anisotropic characteristic between *H*//*ab* and *H*//*c*, which could be well scaled into a universal curve. The peak value $-\Delta {S}_{M}^{{\max }}$ of 1.20 J kg^−1^ K^−1^ and the corresponding adiabatic temperature change Δ*T*_*ad*_ of 0.66 K are deduced from heat capacity with out-of-plane field change of 5 T. By fitting of the field-dependent parameters of $-\Delta {S}_{M}^{{\max }}$ and the relative cooling power RCP, it gives $-{\rm{\triangle }}{S}_{M}^{{\max }}$ ∝ *H*^*n*^ with *n* = 0.603(6) and *RCP* ∝ *H*^*m*^ with *m* = 1.20(1) when *H*//*c*. Given the high and tunable *T*_*c*_, Fe_3−*x*_GeTe_2_ crystals are of interest for fabricating the heterostructure-based spintronics device.

## Introduction

Intrinsic long-range ferromagnetism recently achieved in two-dimensional-limit van der Waals (vdW) crystals opens up great possibilities for both studying fundamental two-dimensional (2D) magnetism and engineering novel spintronic vdW heterostuctures^1–5^. Fe_3_GeTe_2_ is a promising candidate since its Curie temperature (*T*_*c*_) in bulk is high and depends on the concentration of Fe atoms, ranging from 150 to 230 K^6–11^. Intrinsic magnetocrystalline anisotropy in few-layer counteracts thermal fluctuation and favors the 2D long-range ferromagnetism with a lower *T*_*c*_ of 130 K^5^. Most significantly, the *T*_*c*_ can be ionic-gate-tuned to room temperature in few-layers which is of high interest for electrically controlled magnetoelectronic devices^12^.

**Figure 1:**
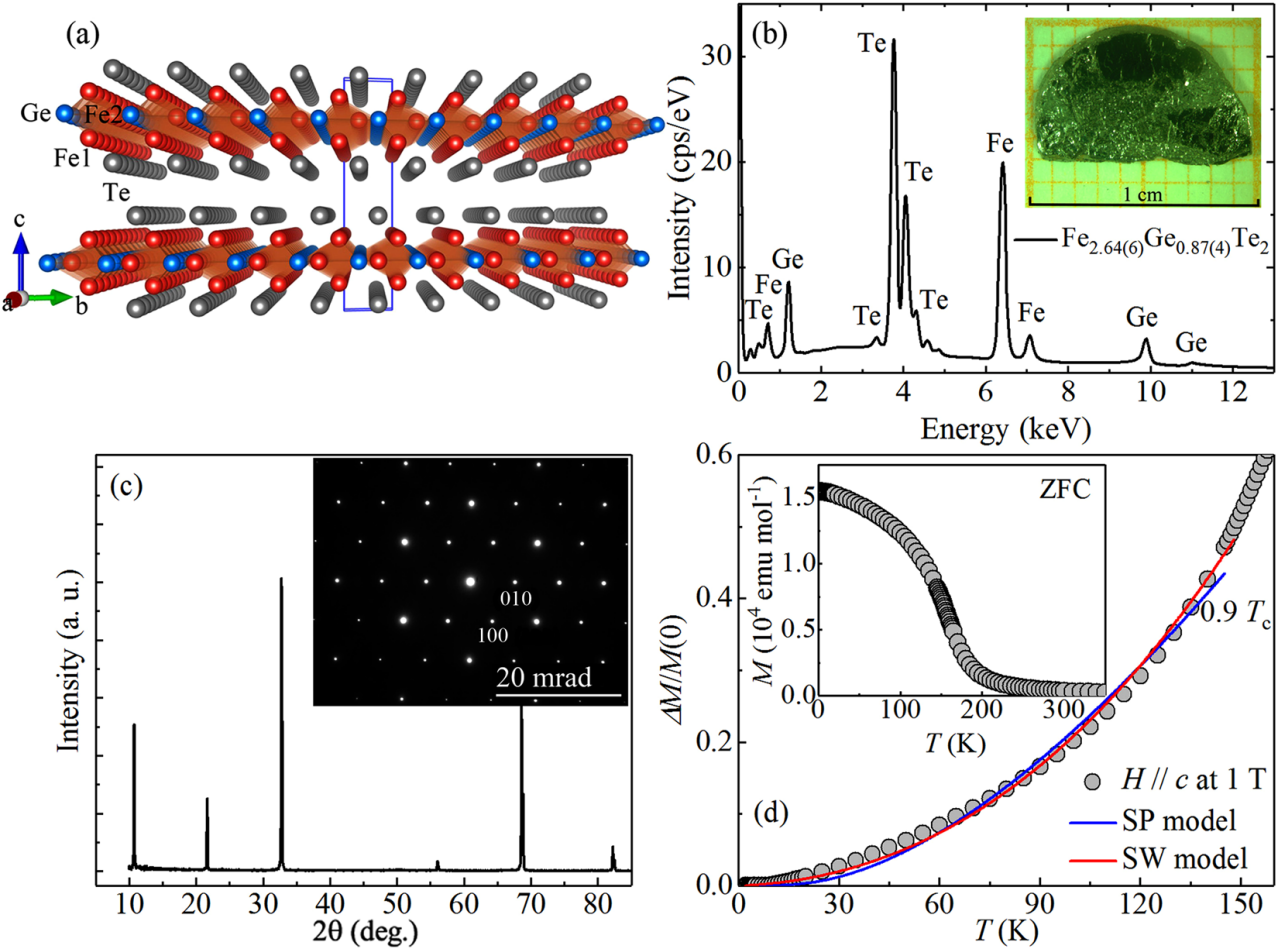
(**a**) Crystal structure and (**b**) X-ray energy-dispersive spectrum of Fe_3−*x*_GeTe_2_ single crystal. Inset shows a photograph of Fe_3−*x*_GeTe_2_ single crystal on a 1 mm grid. (**c**) X-ray diffraction (XRD) pattern of Fe_3−*x*_GeTe_2_. Inset shows the electron diffraction pattern taken along the [001] zone axis direction. (**d**) Temperature dependence of the reduced magnetization with out-of-plane field of Fe_3−*x*_GeTe_2_ fitted using spin-wave (SW) model and single-particle (SP) model. Inset shows the temperature dependence of zero-field-cooling (ZFC) magnetization of Fe_3−*x*_GeTe_2_ measured at *H* = 1 T applied along the *c* axis.

The layered Fe_3−*x*_GeTe_2_ displays a hexagonal structure belonging to the P6_3_/mmc space group, where the 2D layers of Fe_3−*x*_Ge sandwiched between nets of Te ions are weakly connected by vdW bonding [Fig. 1(a)]^6^. There are two inequivalent Wyckoff positions of Fe atoms which are denoted as Fe1 and Fe2. The Fe1-Fe1 dumbbells are situated in the centre of the hexagonal cell in the honeycomb lattice, composed of covalently bonded Fe2-Ge atoms. No Fe atoms occupy the interlayer space and Fe vacancies only occur in the Fe2 sites^13^. Local atomic environment is also studied by the Mössbauer and X-ray absorption spectroscopies^14,15^. Partially filled Fe *d* orbitals results in an itinerant ferromagnetism in Fe_3−*x*_GeTe_2_^16^, which exhibits exotic physical phenomena such as nontrivial anomalous Hall effect^17–19^, Kondo lattice behavior^20^, strong electron correlations^21^, and unusual magnetic domain structures^22,23^. A second-step satellite transition T^*^ is also observed just below *T*_*c*_, and is not fully understood^10,15^.

Here we address the anisotropy in Fe_3−*x*_GeTe_2_ as well as the magnetocaloric effect investigated by heat capacity and dc magnetization measurements. The magnetocrystalline anisotropy is observed to be temperature-dependent. The magnetic entropy change Δ*S*_*M*_(*T*, *H*) also reveals an anisotropic characteristic and could be well scaled into a universal curve. Moreover, the $-\Delta {S}_{M}^{{\max }}$ follows the power law of *H*^*n*^ with *n* = 0.603(6), and the relative cooling power RCP depends on *H*^*m*^ with *m* = 1.20(1).

## Methods

High quality Fe_3−*x*_GeTe_2_ single crystals were synthesized by the self-flux technique^14^. The element analysis was performed using energy-dispersive X-ray spectroscopy (EDX) in a JEOL LSM-6500 scanning electron microscope (SEM). The selected area electron diffraction pattern was taken via a double aberration-corrected JEOL-ARM200F operated at 200 kV. The dc magnetization and heat capacity were measured in Quantum Design MPMS-XL5 and PPMS-9 systems with the field up to 5T.

## Results and Discussion

The average stoichiometry of our flux-grown Fe_3−*x*_GeTe_2_ single crystals was determined by examination of multiple points. The actual concentration is determined to be Fe_2.64(6)_Ge_0.87(4)_Te_2_ [Fig. 1(b)], and it is referred to as Fe_3−*x*_GeTe_2_ throughout this paper. The as-grown single crystals are mirror-like and metallic platelets with the crystallographic *c* axis perpendicular to the platelet surface with dimensions up to 10 millimeters [inset in Fig. 1(b)]. In the 2*θ* X-ray diffraction pattern [Fig. 1(c)], only the (00*l*) peaks are detected, confirming the crystal surface is normal to the *c* axis. The corresponding electron diffraction pattern [inset in Fig. 1(c)] also confirms the high quality of single crystals.

Figure 1(d) presents the low temperature thermal demagnetization analysis for Fe_3−*x*_GeTe_2_ with out-of-plane field using both spin-wave (SW) model and single-particle (SP) model. The temperature dependence of zero-field-cooling (ZFC) magnetization *M*(*T*) for Fe_3−*x*_GeTe_2_ measured in *H* = 1 T applied along the *c* axis is shown in the inset of Fig. 1(d). Localized-moment spin-wave excitations can be described by a Bloch equation^24–26^:

1 $$\frac{\Delta M}{M(0)}=\frac{M(0)-M(T)}{M(0)}=A{T}^{3/2}+B{T}^{5/2}+\cdots ,$$

where *A* and *B* are the coefficients. The *M*(0) is the magnetization at 0 K, which is usually estimated from the extrapolation of *M*(*T*). The *T* ^3/2^ term stems from harmonic contribution and the *T*^5/2^ term is a high-order contribution in spin-wave dispersion relation. In an itinerant magnetism, it is a result of excitation of electrons from one subband to the other. The single-particle excitation is^24^:

2 $$\frac{\Delta M}{M(0)}=\frac{M(0)-M(T)}{M(0)}=C{T}^{3/2}exp\frac{-\Delta }{{k}_{B}T},$$

where *C*, Δ and *k*_*B*_ are fit coefficient, the energy gap between the Fermi level and the top of the full subband and the Boltzmann constant, respectively. It can be seen that the SW model gives a better fit than the SP model up to 0.9 *T*_*c*_ [Fig. 1(d)], indicating possible localized moment, in agreement with the bad-metallic resistivity of Fe_3−*x*_GeTe_2_^15^. It is also understandable that the SP model fails due to strong electron correlation in Fe_3−*x*_GeTe_2_^21^. The fitting yields *A* = 8.4(7) × 10^−5^ K^−3/2^, *B* = 1.24(5) × 10^−6^ K^−5/2^, *C* = 3.4(1) × 10^−4^ K^−3/2^ and Δ = 3.9(4) meV.

**Figure 2:**
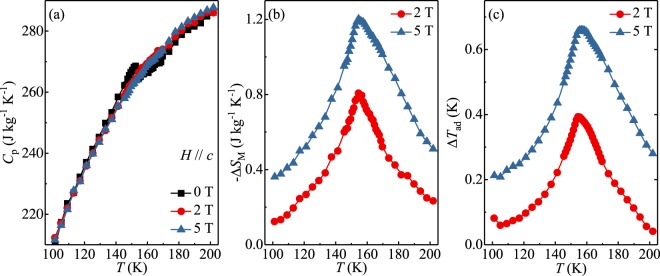
Temperature dependences of (**a**) the specific heat *C*_*p*_, (**b**) the magnetic entropy change −Δ*S*_*M*_, and (**c**) the adiabatic temperature change Δ*T*_*ad*_ for Fe_3−*x*_GeTe_2_ with out-of-plane field changes of 2 and 5T.

Figure 2(a) shows the temperature dependence of heat capacity *C*_*p*_ for Fe_3−*x*_GeTe_2_ measured in zero-field and out-of-plane field of 2 and 5 T, respectively. The ferromagnetic order anomaly at *T*_*c*_ = 153 K in the absence of magnetic field is gradually suppressed in fields. The entropy *S*(*T*, *H*) can be determined by

3 $$S(T,H)={\int }_{0}^{T}\frac{{C}_{p}(T,H)}{T}dT.$$

The magnetic entropy change Δ*S*_*M*_(*T*, *H*) can be approximated as Δ*S*_*M*_(*T*, *H*) = *S*_*M*_(*T*, *H*) − *S*_*M*_(*T*, 0). In addition, the adiabatic temperature change Δ*T*_*ad*_ caused by the field change can be derived by Δ*T*_*ad*_(*T*, *H*) = *T*(*S*, *H*) − *T*(*S*, 0) at constant total entropy *S*(*T*, *H*). Figure 2(b,c) present the temperature dependence of −Δ*S*_*M*_ and Δ*T*_*ad*_ estimated from heat capacity with out-of-plane field. They are asymmetric and attain a peak around *T*_*c*_. The maxima of −Δ*S*_*M*_ and Δ*T*_*ad*_ increase with increasing field and reach the values of 1.20 J kg^−1^ K^−1^ and 0.66 K, respectively, with the field change of 5 T. Since a large magnetic anisotropy is observed in Fe_3−*x*_GeTe_2_, it is of interest to further calculate its anisotropic magnetic entropy change.

**Figure 3:**
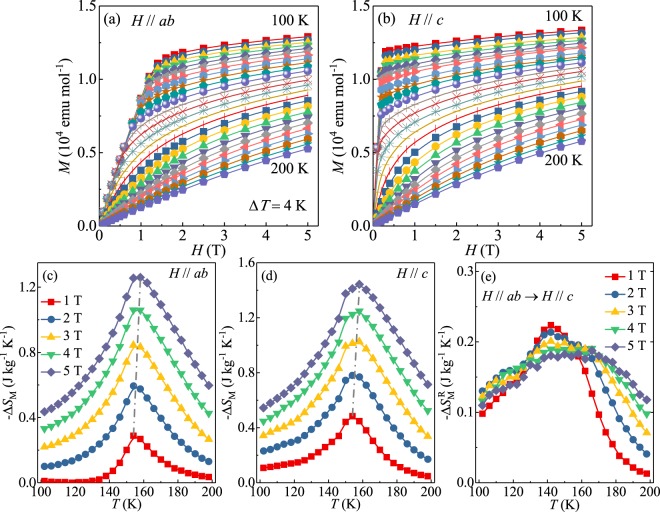
Initial isothermal magnetization curves from *T* = 100 to 200 K with temperature step of *T* = 4 K measured with (**a**) in-plane and (**b**) out-of-plane fields. Temperature dependence of magnetic entropy change −Δ*S*_*M*_ obtained with (**c**) in-plane and (**d**) out-of-plane field changes, and (**e**) the difference −$\Delta {S}_{M}^{R}$.

Figure 3(a,b) present the magnetization isotherms with field up to 5 T applied in the *ab* plane and along the *c* axis, respectively, in temperature range from 100 to 200 K with a temperature step of 4 K. The magnetic entropy change can be obtained from dc magnetization measurement as^27^:

4 $$\Delta {S}_{M}(T,H)={\int }_{0}^{H}{[\frac{\partial S(T,H)}{\partial H}]}_{T}dH.$$

With the Maxwell’s relation ${[\frac{\partial S(T,H)}{\partial H}]}_{T}$ = ${[\frac{\partial M(T,H)}{\partial T}]}_{H}$, it can be rewritten as^28^:

5 $$\Delta {S}_{M}(T,H)={\int }_{0}^{H}{[\frac{\partial M(T,H)}{\partial T}]}_{H}dH.$$

When the magnetization is measured at small temperature and field steps, Δ*S*_*M*_(*T*, *H*) is approximated:

6 $$\Delta {S}_{M}(T,H)=\frac{{\int }_{0}^{H}M(T+\Delta T)dH-{\int }_{0}^{H}M(T)dH}{\Delta T}.$$

Figure 3(c,d) show the calculated −Δ*S*_*M*_(*T*, *H*) as a function of temperature in various fields up to 5 T applied in the *ab* plane and along the *c* axis, respectively. All the −Δ*S*_*M*_(*T*, *H*) curves feature a pronounced peak around *T*_*c*_, similar to those obtained from heat capacity [Fig. 2(b)], and the peak broadens asymmetrically on both sides with increase in field. Moreover, the value of −Δ*S*_*M*_(*T*, *H*) increases monotonically with increase in field; the peak −Δ*S*_*M*_ reaches 1.26 J kg^−1^ K^−1^ with in-plane field change and 1.44 J kg^−1^ K^−1^ with out-of-plane change of 5 T, respectively. We calculated the rotating magnetic entropy change $\Delta {S}_{M}^{R}$ as

7 $$\Delta {S}_{M}^{R}(T,H)=\Delta {S}_{M}(T,{H}_{c})-\Delta {S}_{M}(T,{H}_{ab}).$$

The asymmetry of −Δ*S*_*M*_(*T*, *H*) is more apparent in the temperature dependence of −$\Delta {S}_{M}^{R}$ [Fig. 3(e)]. Furthermore, there is a slight shift of −Δ*S*_*M*_ maximum towards higher temperature when the field varies from 1 to 5 T [Fig. 3(c,d)]. This shift of *T*_*peak*_ excludes the mean field model but could be reproduced by the Heisenberg model due to its discrepancy with *T*_*c*_^29^.

Around the second order phase transition^30^, the magnetic entropy maximum change is $-\Delta {S}_{M}^{{\max }}$ = *aH*^*n*^ ^31^, where *a* is a constant and *n* is^32^

8 $$n(T,H)=dln|\Delta {S}_{M}|/dln(H).$$

**Figure 4:**
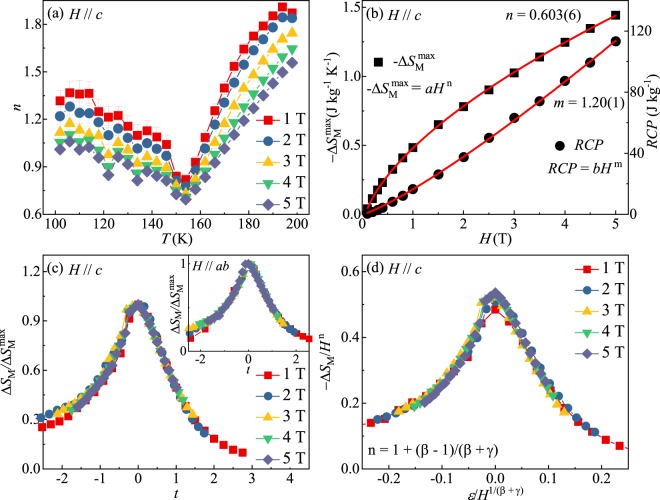
(**a**) Temperature dependence of *n* in various fields. (**b**) Field dependence of the maximum magnetic entropy change $-\Delta {S}_{M}^{{\max }}$ and the relative cooling power RCP with power law fitting in red solid lines. (**c**) The normalized Δ*S*_*M*_ as a function of the rescaled temperature *t* with out-of-plane field and in-plane field (inset). (**d**) Scaling plot based on the critical exponents *β* = 0.372 and *γ* = 1.265^14^.

Figure 4(a) shows the temperature dependence of *n*(*T*) in various fields. All the *n*(*T*) curves follow an universal behavior^33^. At low temperatures, *n* has a value close to 1. At high temperatures, *n* tends to 2 as a consequence of the Curie-Weiss law. At *T* = *T*_*c*_, *n* has a minimum. Additionally, the exponent *n* at *T*_*c*_ is related to the critical exponents^30^:

9 $$n({T}_{c})=1+(\frac{\beta -1}{\beta +\gamma })=1+\frac{1}{\delta }(1-\frac{1}{\beta }),$$

where *β*, *γ*, and *δ* are the critical exponents related to the spontaneous magnetization *M*_*s*_ below *T*_*c*_, the inverse initial susceptibility *H*/*M* above *T*_*c*_, and the isotherm *M*(*H*) at *T*_*c*_, respectively.

Relative cooling power (RCP) could be used to estimate the cooling efficiency^34^:

10 $$RCP=-\,\Delta {S}_{M}^{max}\times \delta {T}_{FWHM},$$

where $-\Delta {S}_{M}^{{\max }}$ is the entropy change maximum around *T*_*c*_ and *δT*_*FWHM*_ is the width at half maximum. The RCP also depends on the field as *RCP* = *bH*^*m*^, where *b* is a constant and *m* is related to the critical exponent *δ*:

11 $$m=1+\frac{1}{\delta }.$$

Figure 4(b) presents the field-dependent $-\Delta {S}_{M}^{{\max }}$ and RCP. The RCP is 113.3 J kg^−1^ within field change of 5 T for Fe_3−*x*_GeTe_2_. This is one half of those in manganites and much lower than in ferrites^35,36^. Fitting of the $-\Delta {S}_{M}^{{\max }}$ and RCP gives *n* = 0.603(6) and *m* = 1.20(1), which are close to the values estimated from the critical exponents (Table 1).

**Table 1:** Critical exponents of Fe_3−*x*_GeTe_2_^14^. The MAP, KFP and CI represent the modified Arrott plot, the Kouvel-Fisher plot and the critical isotherm, respectively.

Technique	*β*	*γ*	*δ*	*n*	*m*
$-\Delta {S}_{M}^{{\max }}$				0.603(6)	
RCP					1.20(1)
MAP	0.374(1)	1.273(8)	4.404(12)	0.620(1)	1.227(1)
KFP	0.372(4)	1.265(15)	4.401(6)	0.616(2)	1.227(1)
CI			4.50(1)		1.222(1)

The scaling of magnetocaloric data is constructed by normalizing all the −Δ*S*_*M*_ curves against the maximum $-\Delta {S}_{M}^{{\max }}$, namely, Δ*S*_*M*_/$\Delta {S}_{M}^{{\max }}$ by rescaling the temperature *t* below and above *T*_*c*_ as defined in:

12 $${t}_{-}=({T}_{peak}-T)/({T}_{r1}-{T}_{peak}),T < {T}_{peak},$$

13 $${t}_{+}=(T-{T}_{peak})/({T}_{r2}-{T}_{peak}),T > {T}_{peak},$$

where *T*_*r*1_ and *T*_*r*2_ are the temperatures of two reference points corresponding to $\Delta {S}_{M}({T}_{r1},{T}_{r2})=\frac{1}{2}\Delta {S}_{M}^{max}$^37^. All the −Δ*S*_*M*_(*T*, *H*) curves collapse onto a single curve regardless of temperature and field, as shown in Fig. 4(c). In the phase transition region, the scaling analysis of −Δ*S*_*M*_ can also be expressed as

14 $$\frac{-\Delta {S}_{M}}{{a}_{M}}={H}^{n}f(\frac{\varepsilon }{{H}^{1/\Delta }}),$$

where *a*_*M*_ = *T*_*c*_^−1^*A*^*δ*+1^*B* with A and B representing the critical amplitudes as in *M*_*s*_(*T*) = *A*(−*ε*)^*β*^ and *H* = *BM*^*δ*^, Δ = *β* + *γ*, and *f*(*x*) is the scaling function^38^. If the critical exponents are appropriately chosen, the −Δ*S*_*M*_(*T*) curves should be rescaled into a single curve, consistent with normalizing all the −Δ*S*_*M*_ curves with two reference temperatures. By using the values of *β* = 0.372 and *γ* = 1.265 obtained by the Kouvel-Fisher plot^14^, we have replotted the scaled −Δ*S*_*M*_ for Fe_3−*x*_GeTe_2_ [Fig. 4(d)]. The good overlap of the experimental data points clearly indicates that the obtained critical exponents for Fe_3−*x*_GeTe_2_ are not only in agreement with the scaling hypothesis but also intrinsic.

**Figure 5:**
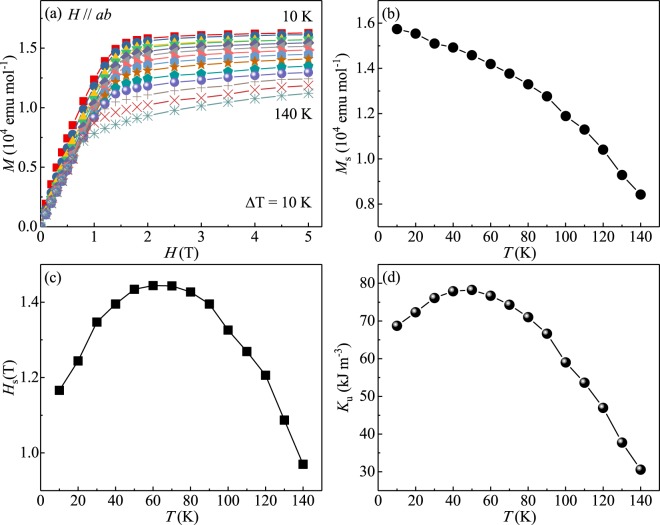
(**a**) Initial isothermal magnetization curves from *T* = 10 to 140 K with in-plane fields. Temperature evolution of (**b**) the saturation magnetization *M*_*s*_, (**c**) the saturation field *H*_*s*_, and (**d**) the anisotropy constant *K*_*u*_.

Then we estimated the magnetocrystalline anisotropy of Fe_3−*x*_GeTe_2_. By using the Stoner-Wolfarth model a value for the magnetocrystalline anisotropy constant *K*_*u*_ can be estimated from the saturation regime in isothermal magnetization curves [Fig. 5(a)]^39^. Within this model the magnetocrystalline anisotropy in the single domain state is related to the saturation magnetic field *H*_*s*_ and the saturation moment *M*_*s*_ with *μ*_0_ is the vacuum permeability:

15 $$\frac{2{K}_{u}}{{M}_{s}}={\mu }_{0}{H}_{sat}.$$

When *H*//*ab*, the anisotropy becomes maximal. We estimated the saturation magnetization *M*_*s*_ by using a linear fit of *M*(*H*) above a magnetic field of 2.5 T with in-plane field [Fig. 5(b)], which monotonically decreases with increasing temperature. Then we determined the saturation field *H*_*s*_ as the intersection point of two-linear fits, one being a fit to the saturated regime at high fields and one being a fit of the unsaturated linear regime at low fields. The value of *H*_*s*_ increases at low temperature, which is possibly related to a spin reorientation transition^15^, and then decreases with increasing temperature [Fig. 5(c)]. Figure 5(d) presents the temperature evolution of *K*_*u*_ for Fe_3−*x*_GeTe_2_, which can not be described by the *l*(*l* + 1)/2 power law^40,41^. The value of *K*_*u*_ for Fe_3−*x*_GeTe_2_ is about 69 kJ cm^−3^ at 10 K, slightly increases to 78 kJ cm^−3^ at 50 K, and then decrease with increasing temperature, which are comparable to those for CrBr_3_, but smaller than those for CrI_3_^42^. The decrease of *K*_*u*_ with increasing temperature is also observed in CrBr_3_ and CrI_3_^42^, arising from a large number of local spin clusters^43,44^. In a pure two-dimensional system, materials with isotropic short-range exchange interactions can not magnetically order. The long-range ferromagnetism in few-layers of Fe_3−*x*_GeTe_2_ could possibly be favored by the large magnetocrystalline anisotropy.

## Conclusion

In summary, we have investigated in detail the magnetocaloric effect of Fe_3−*x*_GeTe_2_ single crystals. The large magnetocrystalline anisotropy is found to be temperature-dependent and probably establishes the long-range ferromagnetism in few-layers of Fe_3−*x*_GeTe_2_. The magnetic entropy change −Δ*S*_*M*_ also reveals an anisotropic characteristic and could be well scaled into a universal curve independent on temperature and field. By fitting of the field-dependent parameters of $-\Delta {S}_{M}^{{\max }}$ and the relative cooling power RCP, it gives $-\Delta {S}_{M}^{{\max }}$∝*H*^*n*^ with *n* = 0.603(6) and *RCP* ∝ *H*^*m*^ with *m* = 1.20(1) when *H*//*c*. Considering its tunable room-temperature ferromagnetism and hard magnetic properties in nanoflakes, further investigation on the size dependence of magnetocaloric effect is of interest.
