# Author Correction: Anisotropic magnetocaloric effect in Fe_3−*x*_GeTe_2_

**DOI:** 10.1038/s41598-020-58154-9

**Published:** 2020-01-28

**Authors:** Yu Liu, Jun Li, Jing Tao, Yimei Zhu, Cedomir Petrovic

**Affiliations:** Condensed Matter Physics and Materials Science Department, Brookhaven National Laboratory, Upton, New York, 11973 USA

Correction to: *Scientific Reports* 10.1038/s41598-019-49654-4, published online 13 September 2019

The Acknowledgements section in this Article is incomplete.

“We thank J. Warren for help with the scanning electron microscopy (SEM) measurement. Work at Brookhaven National Laboratory was supported by US DOE, Office of Science, Office of Basic Energy Sciences under contract DE-SC0012704.”

should read:

“We thank J. Warren for help with the scanning electron microscopy (SEM) measurement. This work was funded by the Computation Material Science Program (Y.L. and C.P.). TEM studies at Brookhaven National Laboratory were supported by US DOE, Office of Science, Office of Basic Energy Sciences under contract DE-SC0012704.”

